# The Alveolin IMC1h Is Required for Normal Ookinete and Sporozoite Motility Behaviour and Host Colonisation in *Plasmodium berghei*

**DOI:** 10.1371/journal.pone.0041409

**Published:** 2012-07-23

**Authors:** Katrin Volkmann, Claudia Pfander, Charlotte Burstroem, Malika Ahras, David Goulding, Julian C. Rayner, Friedrich Frischknecht, Oliver Billker, Mathieu Brochet

**Affiliations:** 1 The Wellcome Trust Sanger Institute, Hinxton, United Kingdom; 2 Department of Parasitology, Hygiene Institute, University of Heidelberg Medical School, Heidelberg, Germany; Museum National d'Histoire Naturelle, France

## Abstract

Alveolins, or inner membrane complex (IMC) proteins, are components of the subpellicular network that forms a structural part of the pellicle of malaria parasites. In *Plasmodium berghei*, deletions of three alveolins, IMC1a, b, and h, each resulted in reduced mechanical strength and gliding velocity of ookinetes or sporozoites. Using time lapse imaging, we show here that deletion of IMC1h (PBANKA_143660) also has an impact on the directionality and motility behaviour of both ookinetes and sporozoites. Despite their marked motility defects, sporozoites lacking IMC1h were able to invade mosquito salivary glands, allowing us to investigate the role of IMC1h in colonisation of the mammalian host. We show that IMC1h is essential for sporozoites to progress through the dermis *in vivo* but does not play a significant role in hepatoma cell transmigration and invasion *in vitro*. Colocalisation of IMC1h with the residual IMC in liver stages was detected up to 30 hours after infection and parasites lacking IMC1h showed developmental defects *in vitro* and a delayed onset of blood stage infection *in vivo*. Together, these results suggest that IMC1h is involved in maintaining the cellular architecture which supports normal motility behaviour, access of the sporozoites to the blood stream, and further colonisation of the mammalian host.

## Introduction

The phylum Apicomplexa contains numerous unicellular parasites that cause severe diseases in humans and other animals. During their life cycle, apicomplexan parasites go through multiple developmental stages and adopt a range of cell shapes, including both motile and non-motile forms. The process of host cell invasion appears to be strongly conserved, as both the basic cell ultrastructure and many of the proteins involved in invasion are conserved amongst apicomplexan species. For example, invasive stages of malaria parasites, as well as related apicomplexan parasites, all possess a unique cortical structure called the pellicle. This structure is made up of the plasma membrane, the inner membrane complex (IMC), subpellicular microtubules and the subpellicular network (SPN). The gliding motion of the parasite is thought to be dependent on the parasite's microtubule network that is twisted slightly around the longitudinal axis of the cell. The SPN corresponds to a network of filaments, situated on the cytoplasmic side of the IMC, which shapes the cell and acts as a membrane skeleton [1], [2], [3].

The IMC lies below the plasma membrane and consists of flattened membrane sacs called alveoli [4]. It serves as support for the actomyosin motor apparatus that is required for motility and host cell invasion and is located between the plasma membrane and the outer layer of the IMC. This motor complex includes the essential class XIV myosin A heavy chain (MyoA), the myosin light chain (myosin tail-interacting protein, MTIP) and is firmly anchored in the plane of the outer IMC by the integral membrane protein GAP50 and the lipid anchored GAP45 [5]. *Plasmodium* PfGAPM1 to 3 (also referred to as PfM6Tα to γ) also localise to the IMC and were shown to co-purify with alveolins and the actomyosin motor itself [6], [7]. Furthermore, in *Toxoplasma gondii* tachyzoites the IMC has been shown to play a critical role in cell division, which is driven by the assembly of the cortical cytoskeleton and serves as a scaffold for organellogenesis and organelle partitioning [8].

Alveolins share a core of repeated sequence motifs, flanked on either side by unique and non-repetitive sequences. The repeated sequences can vary in length and in the number of intervening amino acids, but typically harbour a core sub-repeat being EKIVEVP or very similar. Apicomplexan parasites show a diverse array of alveolins with twelve and seven members of the alveolin family identified in *T. gondii* and *Plasmodium falciparum*, respectively. This diversity could be necessary to fulfil the diverse role of the cytoskeleton in each stage of the life cycle. In agreement with this hypothesis, alveolins studied so far are expressed at different times and perhaps for different purposes [4]. A study in *T. gondii* indicated a key role for TgIMC1 in maintaining cell shape [1]. Recently, the spatio-temporal dynamics of 14 alveolins and alveolin-related proteins was investigated throughout *T. gondii* tachyzoite development. In addition to the IMC some of these proteins were also localising to the basal complex or the centrosome [9].

In *P. berghei*, high-throughput mass spectrometry-based protein analysis of different life cycle stages revealed that five alveolin-related proteins were expressed in ookinetes, while only two members of this family could be detected in sporozoites [10]. IMC1a, the structural orthologue of TgIMC1, is expressed during sporozoite development only. It was shown to be necessary for normal sporozoite cell shape, mechanical stability, normal gliding velocity, and mosquito salivary gland invasion [2]. IMC1b is a closely related structural paralogue of IMC1a expressed in ookinetes only. IMC1b-deficient ookinetes displayed an abnormal cell shape, reduced gliding velocity, decreased mechanical strength, and reduced infectivity of mosquito midgut [11]. Finally, a mutant lacking IMC1h mimicked the loss of function phenotypes of IMC1b and IMC1a in ookinetes and sporozoites, respectively [12].

We have here carried out an independent study on IMC1h (PBANKA_143660 or PbALV3, the orthologue of PfALV3 as defined previously [4]) and confirmed its role in maintaining cell shape, gliding velocity, and infectivity in ookinetes and sporozoites. We have further shown that ookinetes and sporozoites lacking IMC1h displayed marked changes in their motility behaviour. Ookinetes did not show the typical helical gliding but displayed a straight motion pattern while the gliding path of sporozoites was meandering instead of circular. In contrast to what was previously observed for both IMC1a and IMC1h [2], [12], sporozoites lacking IMC1h were detected in the mosquito's salivary glands, although at reduced levels compared to WT. This allowed us to investigate the role of IMC1h in the colonisation of the mammalian host. Colonisation was markedly inhibited in parasites with an IMC1h deletion. IMC1h was essential for sporozoite progression through the dermis *in vivo* and is therefore essential for completing the transmission of the parasite to the host. In contrast, IMC1h did not play a central role in transmigration and invasion of liver cells *in vitro*. However, in early liver stages, IMC1h partially colocalised with the remnant IMC and parasites lacking the protein showed impaired development in hepatoma cells. Together, these results indicate that IMC1h alveolin is required at different stages during the colonisation of the host.

## Results

### IMC1h is detected in ookinetes, sporozoites and early liver stages

To investigate the spatio-temporal expression of IMC1h, we first studied a mutant in which the endogenous gene was fused in frame at its 3′ end to a triple hemagglutinin (HA) epitope tag ([13] and Figure S1). The tagged mutant, which also expresses GFP constitutively to facilitate phenotyping [14], developed normally in mice and mosquitoes and was readily transmitted by mosquito bite, demonstrating that the addition of the HA tag to the carboxyl terminus of the protein did not adversely affect parasite development.

**Figure 1:**
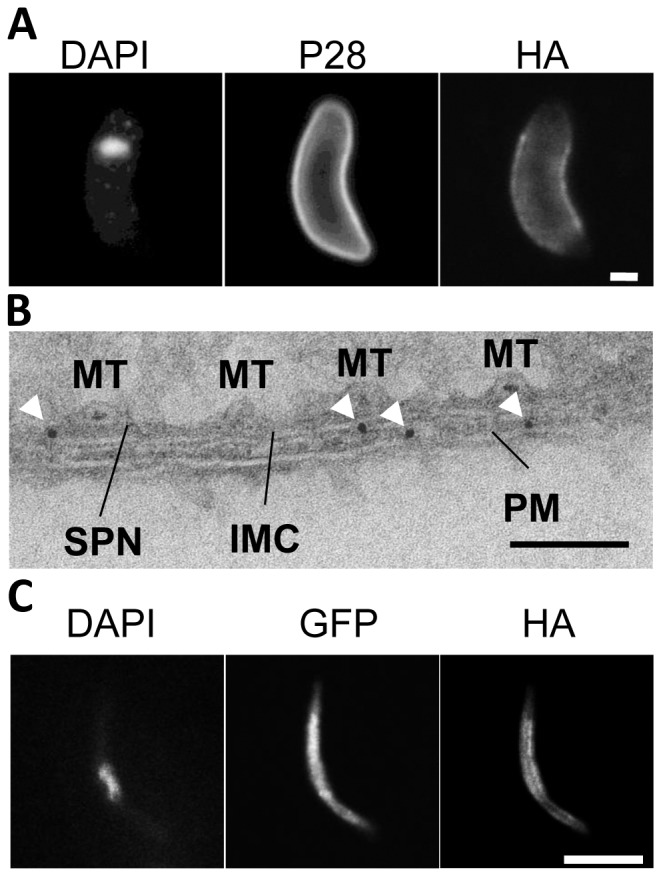
Localisation of IMC1h in ookinetes and sporozoites. A. Single optical section of a mature IMC1h-HA ookinete immunostained for HA, P28 and the nucleus stained with Hoechst. The HA tagged IMC1h localizes to the peripheral edge of the ookinete. Both the anterior and posterior ends are spared by IMC1h-HA whereas P28 covers the whole surface of the ookinete. Scale bar = 1 µm. B. Representative TEM section of an IMC1h-HA ookinete immunogold-labelled for HA showing the pellicle. Gold particles (white arrowheads) localise at the IMC. IMC = inner membrane complex, MT = microtubules, SPN = subpellicular network, PM = plasma membrane. Scale bar = 100 nm. C. Salivary gland IMC1h-HA sporozoite immunostained for HA and GFP. The nucleus is stained with Hoechst. Single optical section is shown revealing the localisation of IMC1h-HA to the cell periphery. The IMC1h-HA is distributed over the full length of the sporozoite. Scale bar = 5 µm.

To follow the subcellular localisation of IMC1h, its expression in asexual blood stages, gametocytes, ookinetes and sporozoites was examined by immunofluorescence microscopy. No fluorescence could be detected in asexual stages and gametocytes. HA-tagged IMC1h localised at the periphery of ookinetes but was absent from both the anterior and posterior ends of the cells (Figure 1A). Immunogold labelling in sections of ookinetes confirmed that IMC1h localised at the IMC (Figure 1B). In salivary gland sporozoites HA immunostaining was present in the cell periphery (Figure 1C).

**Figure 2:**
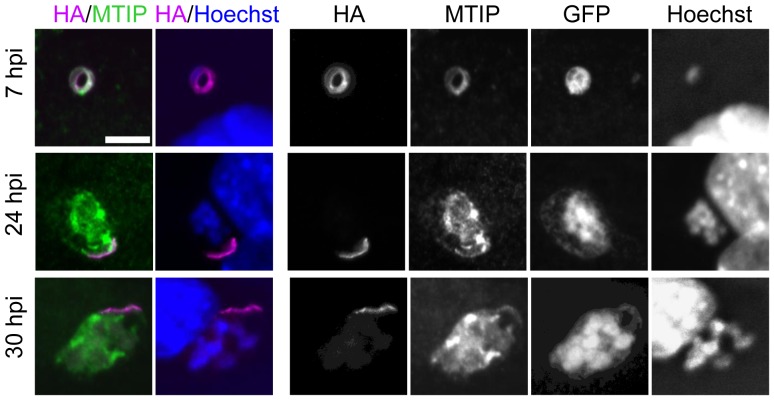
IMC1h-HA localisation in liver stages. Confocal images of intracellular parasites in Hepa 1–6 cells (7, 24, and 30 hpi) after immunofluorescence staining for HA, MTIP, GFP and DNA staining with Hoechst. At 7 hpi IMC1h-HA colocalises with MTIP lining the IMC of the transforming sporozoite. At 24 hpi IMC1h-HA is restricted to a narrow streak at the very edge of the parasitophorous vacuole and still colocalises with MTIP. This area is further reduced at 30 hpi. Scale bar = 5 µm.

To find out whether IMC1h remains detectable during parasite development in the liver, we performed an immunofluorescence assay with antibodies against HA and the IMC associated MTIP protein [15] throughout liver stage development (Figure 2). We observed staining for IMC1h-HA at 7, 24 and 30 hpi. The staining was restricted to the periphery in the transforming sporozoite at 7 hpi as indicated by colocalisation with MTIP. At 24 and 30 hpi the localisation of IMC1h was confined to a peripheral streak which showed partial overlap with MTIP. The streak was observed in all examined infected cells as determined by GFP fluorescence indicating that the signal did not correspond to untransformed sporozoites. From 42 hpi, IMC1h-HA staining had diminished and was absent from mature merozoites in merosomes at 68 hpi while MTIP was present throughout development and localised to the newly formed IMC at the periphery of individual merozoites (data not shown). These results indicate that IMC1h persists in early liver stage parasites, where it shows a highly specific localisation, probably to the residual IMC that is left over from the transforming sporozoite.

### IMC1h-KO ookinetes undergo abnormal development

To study the function of IMC1h and its contribution to parasite development, a genetically modified *P. berghei* parasite, IMC1h-KO, was used in which the complete *imc1h* coding sequence was removed ([13] and Figure S1). To allow a better characterisation of mosquito and liver stages, we also used a parental line constitutively expressing GFP [14]. IMC1h-KO parasites developed normally in mice and were morphologically indistinguishable from wild type (WT) parasites in Giemsa-stained blood films (data not shown). Gametocytes formed and normal exflagellation was observed *in vitro* (data not shown).

**Figure 3:**
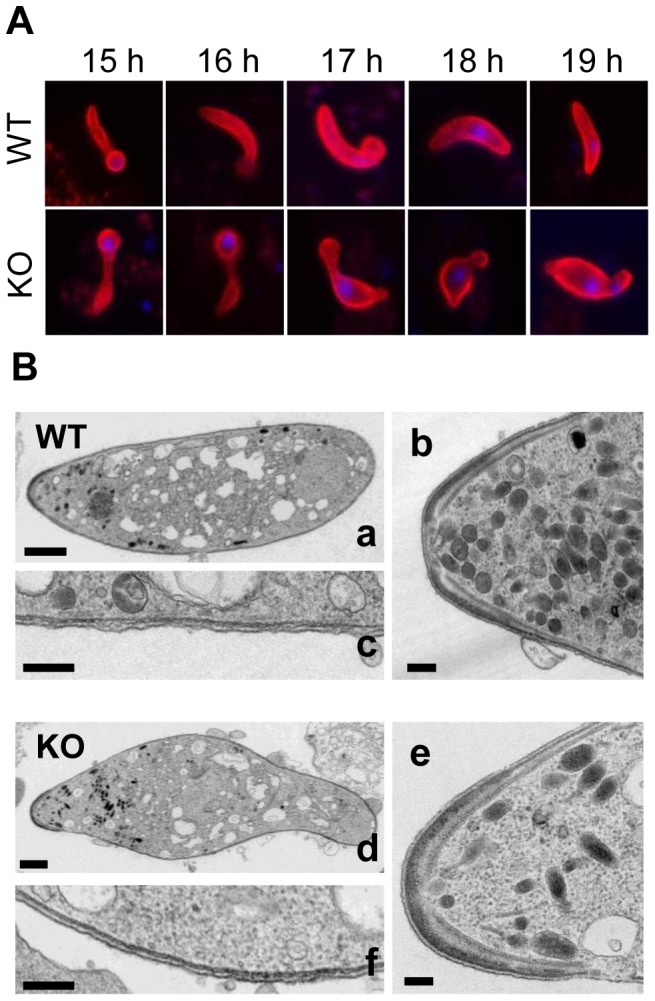
Ookinete morphology of IMC1h-KO. A. Comparative developmental time course from 15 h to 19 h post-fertilisation of WT ookinetes (upper panels) and IMC1h-KO ookinetes (lower panels). Developing ookinetes have been stained for P28 (red) and the nucleus has been counterstained with DAPI (blue). At 16 h, WT ookinetes have transformed to an elongated cell but IMC1h-KO ookinetes failed to reach this even shape. Instead they formed a pronounced bottleneck between the zygote body and the outgrowing protrusion resulting in a widening at the anterior end. B. Longitudinal TEM section of WT (upper panel) and IMC1h-KO (lower panel) ookinetes with anterior end to the left. Low magnification views (a and d) show the abnormal shape of IMC1h-KO (scale bar = 1 µm). No obvious alterations at the apical end (b and e) and the pellicle (c and f) could be observed in higher magnification views (scale bar = 200 nm).

The development of IMC1h-KO ookinetes was investigated *in vitro* (Figure 3A). In the initial 16 hours, development appeared to be normal. However, advanced retort-forms showed abnormal development as the protruding area swelled up leaving a bottleneck between the latter and the remnant “zygote body”. This bottleneck did not prevent the migration of the nucleus leading to ookinetes that were typically wider than WT ookinetes with a bulging area mostly in the anterior part of the cell. None of the mutant ookinetes were found to display the typical banana shape of WT ookinetes. In our ultrastructural examination of purified parasites, IMC1h-KO ookinetes were less homogeneous in shape and size than their WT counterparts. In particular most IMC1h-KO ookinetes rounded up during sample processing, probably due to a loss in cell rigidity (data not shown). Apart from the global shape abnormality, IMC1h-KO ookinetes appeared to possess a complete set of organelles and the assembly of the subpellicular microtubule and IMC appeared unaffected by the gene disruption (Figure 3B).

### IMC1h-KO ookinetes show abnormal gliding behaviour and invasion defects

**Figure 4:**
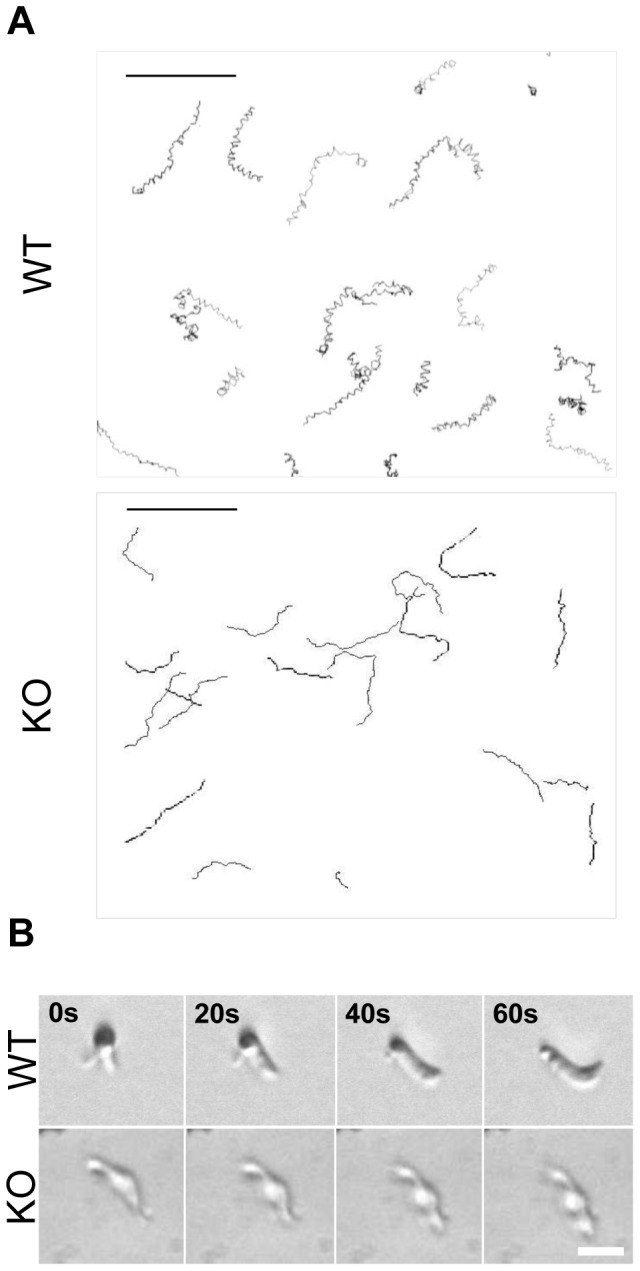
Reconstruction of WT and IMC1h-KO ookinete paths in Matrigel. A. Reconstruction of movement patterns from time-lapse movies of WT (upper panel) and IMC1h-KO ookinetes (lower panel) in Matrigel (20 minutes, one frame every 20 seconds). The reconstructions reveal the classical helical forward movements of gliding WT ookinetes. In contrast the IMC1h-KO ookinetes display a straight forward movement. Scale bar = 50 µm. B. Details of ookinete motility showing drastic shape changes in moving IMC1h-KO ookinetes highlighting the loss of cell rigidity in the mutant. WT ookinetes also showed a 3D exploratory behaviour which was not observed in IMC1h-KO ookinetes. Scale bar = 5 µm.

In order to assess how their abnormal shape could affect motility behaviour, IMC1h-KO ookinetes were enclosed in Matrigel and imaged by time-lapse video microscopy. We determined the average speed by individual cell tracking and distinguished two different movement modes: helical gliding and straight gliding. WT ookinetes moved at an average speed of 6.8±1.2 µm/min, as described previously [16]. IMC1h-KO ookinetes were gliding at a significantly reduced average speed of 3.0±0.92 µm/min (p<0.01). However, a dramatic change in motility behaviour was observed. While 84% of motile WT ookinetes showed a characteristic helical gliding, all IMC1h-KO ookinetes were characterised by a straighter movement (Figure 4A). Upon closer examination, drastic shape changes and constrictions were observed in moving IMC1h-KO ookinetes highlighting the loss of cell rigidity (Figure 4B).

To quantify the ability of IMC1h-KO ookinetes to invade the mosquito midgut epithelium, mosquitoes were fed on infected mice and analysed for ookinete formation in the blood meal and for midgut epithelium invasion. The number of ookinetes in the blood meal was similar in both WT and IMC1h-KO infected mosquitoes suggesting that survival of the mutant is not affected despite its reduced strength. However, a 15-fold decrease in ookinetes that penetrated the midgut epithelium was observed in mosquitoes infected with IMC1h-KO parasites (Table 1). This demonstrated a strongly reduced ability of IMC1h-KO ookinetes to complete the invasion process.

**Table 1:** Parasite development within the mosquito host and transmission at 2, 10 and 21 days post-infection (DPI).

			WT	IMC1h-KO
2 DPI	Ookinetes in blood meal	Conversion rate	**64**±3%	**68**±3%
		Mean number per midgut	**995**±338	**1,040**±293
	Midgut epithelium ookinetes^a^	Prevalence	**10/10** (100%)	**9/10** (90%)
		Mean number per midgut	**268**±86	**18**±9
	Midgut oocysts	Prevalence	**10/10** (100%)	**9/10** (90%)
		Mean number per midgut	**548**±156	**23**±8
10 DPI	Midgut oocysts	Prevalence	**20/20** (100%)	**33/40** (83%)
		Mean number per midgut	**757**±145	**33**±12
21 DPI	Midgut sporozoites	Prevalence	**36/36** (100%)	**69/84** (82%)
		Mean number per midgut	**110,408**±12,315	**20,168**±6,161
	Salivary gland sporozoites	Prevalence	**57/72** (79%)	**89/163** (54%)
		Mean number per salivary gland	**7,912**±1,113	**380**±258
	Transmission	No. infected/no. mice fed on	**3/3**	**0/3**
		No. mice infected/no. mice injected sc^a^	**4/4**	**0/4**
		No. mice infected/no. mice injected iv^b^	**3/3**	**3/3**

a 10,000 salivary gland sporozoites were injected per mouse subcutaneously.

b 10,000 salivary gland sporozoites were injected per mouse intravenously.

### IMC1h-KO oocysts develop normally but form abnormal sporozoites

**Figure 5:**
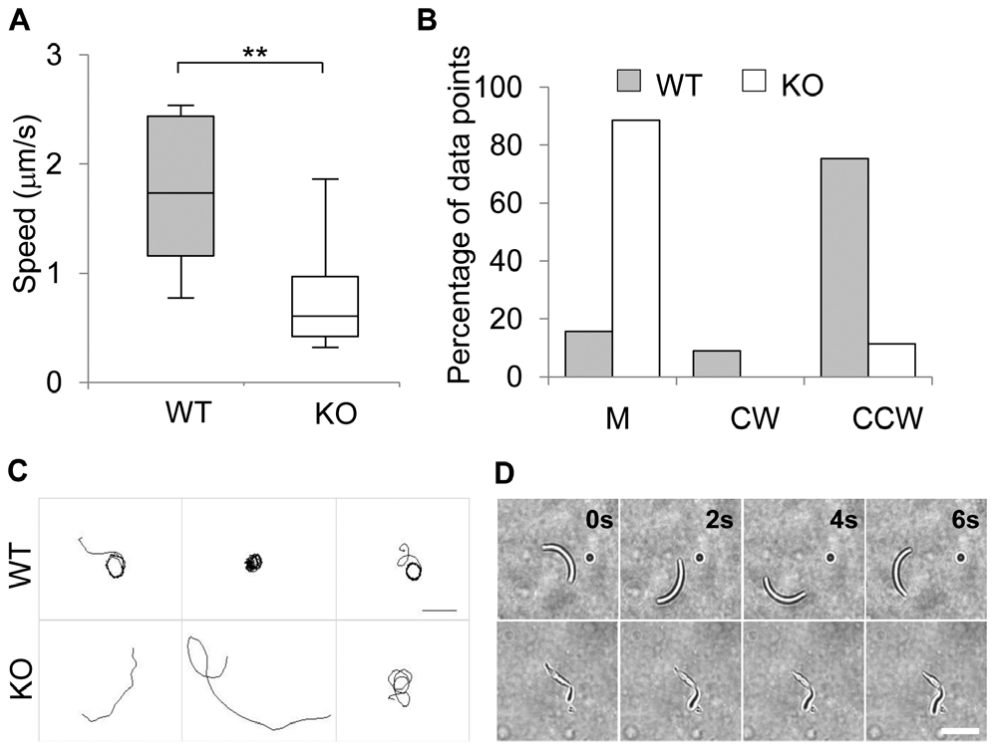
Characteristics of sporozoite motility in Matrigel. A. Speed of WT (n = 15) and IMC1h-KO (n = 11) sporozoites. B. Results of the manual assignment of movement patterns of WT (n = 15) and IMC1h-KO (n = 11) sporozoites. M = Meandering, CW = clock-wise, and CCW = counter clock-wise. C. Reconstruction of tracked sporozoite paths from high-magnification movies revealing movement patterns (two frames per second for 100 s). WT sporozoites (upper panel) mainly display a circular mode of motility while IMC1h-KO sporozoites (lower panel) mainly show a meandering mode of motility. Scale bar = 20 µm. D. Brightfield time-lapse image sequences showing motile WT and IMC1h-KO sporozoites. Scale bar = 10 µm.

The number of IMC1h-KO oocysts in infected mosquitoes was 20-fold lower than in mosquitoes infected with WT parasites (Table 1). Mutant ookinetes that reached the basal epithelium developed into oocysts of normal size and morphology that gave rise to sporozoites. Sporozoites were detected in mosquito salivary glands, which was not reported in a previous characterisation of IMC1h mutant sporozoites [12]. This discrepancy may be explained by a higher mosquito colonisation rate observed in our experimental conditions and by the use of a parasite line constitutively expressing GFP facilitating parasite detection. However, assuming a linear relationship between midgut and salivary gland sporozoite numbers, midgut IMC1h-KO sporozoites were four times less invasive than WT sporozoites (Table 1). All IMC1h-KO sporozoites obtained from both oocysts (data not shown) and salivary glands (Figure 5D) had an abnormal shape, with a protruding bulge of variable size at the posterior end containing the nucleus.

### IMC1h-KO sporozoites show gliding motility defects

Sporozoites rely on their own motility to reach and enter host cells. We thus investigated the motility of IMC1h-KO salivary gland sporozoites (Figure 5) by time-lapse video microscopy in Matrigel. We manually assigned the mode of sporozoite movement as previously described [17], [18]: attached, meandering, waving, gliding in a counter clock-wise (CCW) direction, and gliding in a clock-wise (CW) direction.

The average WT sporozoite speed of 1.7 µm/s (Figure 5A) was similar to that described on polystyrene [17]. They were found to mainly glide in an apparent CCW circular fashion (75%), while a smaller percentage (9%) was found to move in the other direction (Figure 5B and 5C). Interestingly, we did not detect attached or waving parasites previously described on a glass substrate [17] but instead observed a meandering movement for 16% of sporozoites. These differences could likely be explained by the different environments provided by Matrigel embedment and a 2D polystyrene substrate, respectively. Although Matrigel provides a 3D substrate, WT sporozoites did not show a strong 3D exploration movement as most of them stayed in focus for the length of the acquisition time.

IMC1h-KO sporozoites were readily moving albeit at a significantly reduced average speed of 0.8 µm/s (Figure 5A). Constrictions going along the surface of the mutant sporozoites were also occasionally observed. However, IMC1h-KO sporozoites exhibited altered motility behaviour as they mainly displayed meandering gliding (89%), while CCW circular gliding only represented 11% of the movement mode (Figure 5B to 5D). In addition, IMC1h-KO sporozoites showed a 3D exploration behaviour as most of them did not stay in focus for the length of the acquisition time (data not shown).

### IMC1h-KO sporozoites are able to invade salivary glands and hepatocytes but are not able to progress through the dermis *in vivo*

The ability of parasite-infected mosquitoes to establish new infections in mice was then examined by allowing mosquitoes to feed on three naive mice 21 days after the initial feed. IMC1h-KO sporozoites were not able to infect mice after mosquito bites while WT parasites were readily observed four days after the feed in all mice (Table 1). Absence of transmission could be explained by the observed low number of mutant sporozoites reaching the salivary glands, and finally the open salivary ducts. As a consequence, the releasable pool of sporozoites might not have been sufficient enough to allow transmission when the mosquito salivates. To bypass this potential bottleneck and achieve similar inoculation levels, we injected 10,000 sporozoites isolated from salivary glands subcutaneously into the scruff of four naive TO mice each for WT and IMC1h-KO parasites (Table 1). Again, none of the IMC1h-KO injected mice became infected, while all mice injected with WT sporozoites showed blood stage infection at day six post injection. This observation suggests that the failure of IMC1h-KO transmission is not only caused by a reduced number of mosquito injected sporozoites.

To define further the step at which the infection failed, we injected 10,000 salivary gland sporozoites into three naive mice each, but this time intravenously (Table 1). Infected erythrocytes were observed in all mice injected with WT or IMC1h-KO sporozoites after a prepatent period of four and five days, respectively. As more mosquito material had to be used to isolate IMC1h-KO sporozoites, we confirmed that sporozoite purification from a tenfold increase in mosquito material did not contribute to the observed delay in patency (data not shown). Genotyping by PCR confirmed transmission of the IMC1h-KO parasite (data not shown). These data showed that mutant sporozoites were able in principle to reach, invade and develop in host hepatocytes once in the bloodstream, but suggested a crucial role of IMC1h in allowing the parasite to progress through the dermis.

### IMC1h-KO sporozoites are able to transmigrate and invade hepatoma-cells *in vitro* but show developmental defects

**Figure 6:**
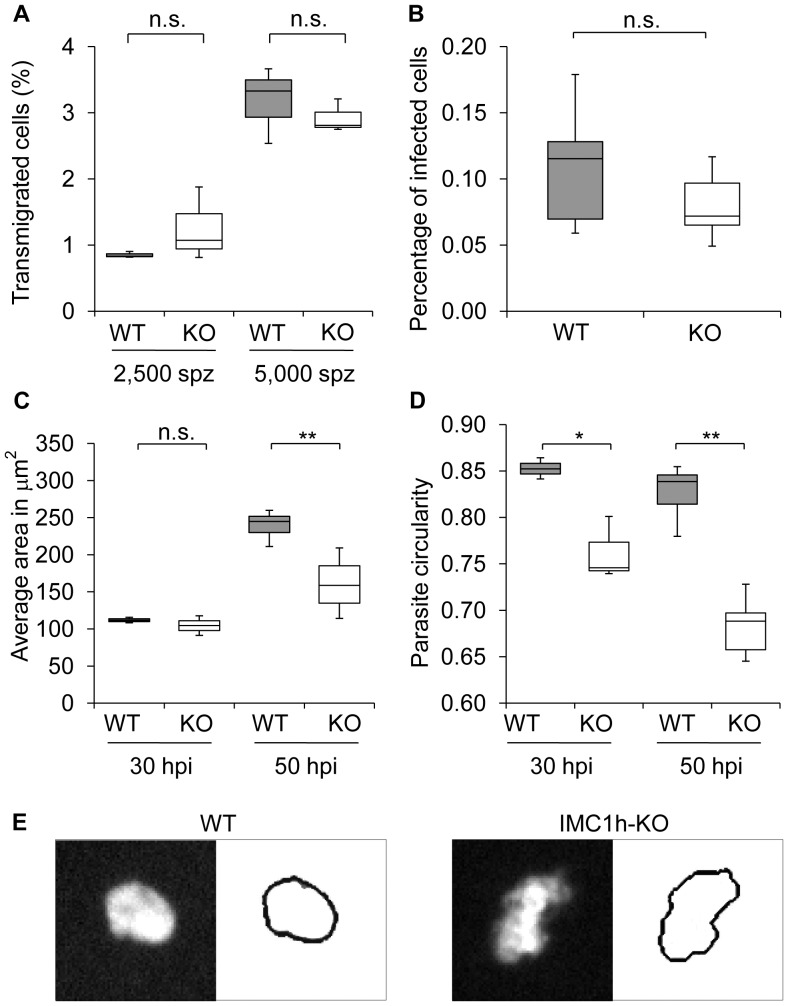
*In vitro* transmigration ability, infectivity and intracellular development of IMC1h-KO parasites. A. Hepa 1–6 cells were incubated with WT or IMC1h-KO sporozoites (spz) in the presence of rhodamine-dextran for two hours. The results are given as the percentage of cells filled with rhodamine-dextran (WT 2,500 spz, n = 3, 257 cells; IMC1h-KO 2,500 spz, n = 3, 234 cells; WT 5,000 spz, n = 3, 561 cells; IMC1h-KO 5,000 spz, n = 3, 403 cells). B. Hepa 1–6 cells invasion rate of WT and IMC1h-KO parasites at 50 hpi. Results are given as percentage of infected cells (WT, n = 6, 341 cells; IMC1h-KO, n = 6, 186 cells). C. Parasite area of WT and IMC1h-KO parasites in Hepa 1–6 cells at 30 and 50 hpi (WT 30 hpi n = 3, 181 cells; IMC1h-KO 30 hpi, n = 3, 99 cells; WT 50 hpi, n = 6, 341 cells; IMC1h-KO 50 hpi, n = 6, 186 cells). D. Average circularity of WT and IMC1h-KO parasites in Hepa 1–6 cells at 50 hpi. Mutant parasites displayed a lower circularity than WT parasites indicating a more irregular and elongated shape (WT 30 hpi n = 3, 181 cells; IMC1h-KO 30 hpi, n = 3, 99 cells; WT 50 hpi, n = 6, 341 cells; IMC1h-KO 50 hpi, n = 6, 186 cells). E. Cropped images acquired with Cellomics ArrayScan VTI HCS Reader show WT (left panel) and IMC1h-KO (right panel) GFP stained parasites. The parasite outline calculated by the SpotDetector BioApplication algorithm is depicted to the right of each image.

Sporozoites traverse cells effectively (Mota et al., 2002) and rely on this ability to reach capillaries within the dermis (Amino et al., 2008). To find out whether the altered motility behaviour of IMC1h-KO sporozoites has an effect on the ability to migrate through cells we performed an *in vitro* transmigration assay in which parasites do not rely on long distance motility to reach cells. We incubated a monolayer of Hepa 1–6 mouse hepatoma-cells with IMC1h-KO or WT sporozoites in the presence of rhodamine-dextran. After quantification of dextran-positive cells we found that IMC1h-KO sporozoites transmigrated with a rate similar to WT sporozoites (Figure 6A).

The detection of blood stage infection was delayed for one day after intravenous injection of IMC1h-KO sporozoites compared to WT. Such a delay in the prepatent period is thought to correspond to a tenfold reduction in parasite load in the liver [19]. To dissect this observation further we analysed the infectivity *in vitro*. IMC1h-KO and WT sporozoites were incubated with Hepa 1–6 cells and analysed for infection rate at 50 hpi. We could not observe a significant difference in infection rate of WT and mutant parasites 50 hpi (Figure 6B) suggesting that IMC1h does not have a crucial role in hepatoma-cell invasion *in vitro*.

As no significant difference could be detected in transmigration and infectivity we further investigate the development of WT and mutant sporozoites by measuring their size and shape at 30 and 50 hpi. Interestingly, the size of the IMC1h-KO exo-erythrocytic forms was significantly reduced compared to WT at 50 hpi but not at 30 hpi (Figure 6C). IMC1h-KO parasites also displayed an atypical shape both at 30 hpi and 50 hpi and this phenotype became more pronounced at 50 hpi (Figure 6D and 6E). The average WT parasites showed a typical smooth oval shape while IMC1h-KO parasites displayed a more irregular shape as reflected by a decrease in circularity. In order to ascertain that these liver stage phenotypes were not due to other secondary mutations in the genome backbone we generated an independent mutant, IMC1h-KO2, and determined the infection rate, size and circularity of this latter. Results obtained with IMC1h-KO2 were similar to those obtained with IMC1h-KO (Figure S2) confirming that the observed phenotypes were due to the modification of the *imc1h* locus. Together these results suggest that defects associated with the absence of IMC1h lead to impaired development in the liver stages *in vitro*.

## Discussion

In this study, we have confirmed that the alveolin IMC1h plays a central role in maintaining cell shape, mechanical strength and infectivity of ookinetes or sporozoites as previously described for IMC1a, IMC1b and IMC1h [2], [11], [12], but have also highlighted that IMC1h has a role in maintaining directionality and gliding behaviour in both these motile stages. In addition we have shown for the first time that an alveolin is required for normal early liver stage development suggesting that the residual IMC still plays a role in this non-motile stage.

Gliding in motile apicomplexan parasites relies on an actin-myosin motor, which is placed just beneath the plasma membrane and is linked to the substrate by transmembrane proteins. Many components that constitute the core of the gliding machinery are known but the underlying mechanisms and the molecular interactions that power efficient locomotion remain still unclear. Studies on malaria parasites and *T. gondii* revealed that the proteins of the myosin motor are anchored into the IMC. What then is the role of the IMC in general, and alveolins in particular, in regulating motility? It was recently proposed that in *P. berghei* ookinetes and sporozoites, alveolins operate autonomously and participate in motility by directly interacting with the actin-myosin motor, independent of their role in maintaining cell shape [12]. This was based on the observation that the absence of both IMC1h and IMC1b did not further affect ookinete shape but further reduced velocity compared with respective single mutants. On the cytoplasmic side of the IMC, a complex filamentous SPN faces the microtubule network, which is twisted slightly around the longitudinal axis. This supra-structure constitutes a very stable structure that displays a remarkable mechanical strength and might serve to confer the crescent shape and the helical gliding motion of ookinetes and sporozoites. It was recently shown that microtubules are connected to the IMC by long linker molecules [3] and alveolins were shown to localise in this filamentous network in *T. gondii* [1]. Parasites lacking IMC1h did not show a characteristic crescent shape and gliding, but displayed a straight gliding although no particular defect in the microtubule organisation could be observed by electron microscopy. This suggests that absence of IMC1h may weaken the complex link between the motor complex and the microtubule network that not only allows cell rigidity, but also the cellular architecture potentially conferring the characteristic movement patterns of ookinetes and sporozoites. 3D tomography and molecular interaction studies would be crucial to investigate this possibility further.

In addition to the striking impact on gliding behaviour and directionality, our *in vivo* experiments suggest that IMC1h-KO ookinetes and sporozoites are strongly impaired in mosquito midgut epithelium and mammalian skin transversal, respectively. There could be multiple causes of this phenotype. Ookinetes and sporozoites are known to undergo considerable constrictions as they migrate [20], [21], and a lack of cell rigidity may directly hinder mosquito midgut epithelium and mammalian skin transversal. We have been able to observe pieces of the posterior end of IMC1h-KO ookinetes detaching from the cell body in Matrigel motility assays (data not shown), highlighting how environmental constraints affect their weakened structure. It was recently shown that structural constraints can be sufficient to guide *Plasmodium* sporozoites in complex environments as 50% of the observed parasites turn in circles in a non-constrained environment while meandering movement increased from 5% to 50% when the complexity of the environment was increased [18]. In light of this work, one could speculate that IMC1h-KO sporozoites are defective in detecting or responding to environmental complexity. Further motility assays with differential environmental constraints should be performed to address this question. IMC1h-KO sporozoites might also be more prone to be attacked by the host immune system due to their decreased gliding speed [22], [23].

The detection of blood stage infection was delayed for one day when the skin passage is bypassed by injecting sporozoites intravenously. This delay could be attributed to the altered motility capacities and an initial delay in liver infection. However it was recently shown that fast gliding motility was not necessary to achieve normal infection after intravenous injections [24]. In addition, in our *in vitro* assay, IMC1h-KO parasites did not display major defects in cellular transmigration or invasion of cultured hepatoma-cells despite their motility defects but showed altered cell size and shape at 50 hpi. These developmental defects might be explained by sub-optimal invasion due to impaired intracellular motility of IMC1-KO as it was previously shown that sporozoites which fail to position themselves in close proximity to the host cell nucleus develop less well [25]. Interestingly, the size of the IMC1h-KO parasites in Hepa 1–6 cells was not different from their WT counterpart up to 30 hpi and IMC1h was detected up to 30 hours after Hepa 1–6 cell invasion. Remodelling of viable sporozoites into liver trophozoites was shown to include dismantlement of the IMC buta residual IMC persists as a retracting network confined to the parasite periphery in early liver stages [26]. We have observed partial colocalisation of IMC1h with MTIP up to 30 hpi suggesting that the organisation of the IMC and the SPN is at least partially conserved up to this time point and plays an unknown role in early liver stage development. Actin polymerisation in hepatocytes shows highly dynamic reorganisation around parasites and generates forces that may cause parasite deformation and disappearance [27]. Absence of IMC1h in liver stage parasites may alter cell rigidity, as shown for IMC1h-KO ookinetes and sporozoites, making the parasites more susceptible to forces generated by host actin polymerisation. It is thus tempting to speculate that IMC1h could play an unknown role during intracellular development in the liver, supporting the possibility that some *Plasmodium* alveolins could have functions in non-invasive life cycle stages as previously suggested for *P. falciparum* gametocytes [4].

In this study we have shown that IMC1h is involved in maintaining the cellular architecture supporting typical movement behaviour of both ookinetes and sporozoites and plays a role at different stages during the colonisation of the mammalian host. Molecular interaction studies and 3D tomography will be necessary to further confirm and dissect the role of alveolins in each of these processes.

## Materials and Methods

### Parasite maintenance, culture, and purification

All animal work was conducted under a license issued by the UK Home Office in accordance with national and international guidelines. Parasites were maintained in female phenyl hydrazine-treated Theiler's Original (TO) outbred mice as described previously [16] and infections monitored on Giemsa-stained blood films. Ookinetes were produced *in vitro* by culturing gametocyte-infected mouse blood in ookinete medium (RPMI1640 containing 25 mM HEPES (Sigma), 10% FCS, 100 µM xanthurenic acid, pH 7.5) and conversion assays were performed by live staining of ookinetes and activated macrogametes with Cy3-conjugated 13.1 monoclonal antibody against P28. The conversion rate was determined as the number of ookinetes as a percentage of the total number of Cy3-fluorescent cells [28].

For transmission experiments batches of ca. 50 female *A. stephensi*, strain SD500, mosquitoes were allowed to feed on mice three days after intraperitoneal injection of infected blood. Unfed mosquitoes were removed the day after, and mosquitoes were maintained on fructose at 19°C. To determine the number of ookinetes in the midgut epithelium, dissected midguts were opened and washed with PBS using 26G needles two days after infection. Oocysts were counted on dissected midguts ten days after feeding. Sporozoite numbers were determined on day 10 and 21 by homogenising midguts and dissected salivary glands and counting the released sporozoites. To determine sporozoite infectivity to mice infected mosquitoes were allowed to feed on naïve TO mice 21 days after infection. Mice were then monitored daily for blood stage parasites.

### Intravenous and subcutaneous injection of sporozoites

WT and mutant sporozoites were freshly isolated in RPMI 1640 containing 1% penicillin/streptomycin (pen/strep) (all Gibco) from 21 day infected *A. stephensi* mosquito salivary glands after dissection. 10,000 WT or IMC1h-KO sporozoites were injected in a volume of 100 µl into the tail vein or mixed with BSA (bovine serum albumin) to a final concentration of 3% and injected in a volume of 100 µl subcutaneously into the scruff of TO mice. Parasitaemia was monitored daily by tail blood smears and Giemsa staining.

### Generation and genomic analysis of genetically modified parasites

All mutants were generated and their genotypes confirmed as part of an effort to establish a scalable method for the production of gene targeting vectors by phage recombineering in *Escherichia coli* [13]. Correct integration of the targeting constructs and expression of the protein were verified by field inversion gel electrophoresis [13], PCR, Western and Southern blotting (Figure S1 and Table S1).

### Immunofluorescence staining, confocal and electron microscopy

Sporozoite and ookinete GFP/P28/HA immunofluorescence assays were performed as previously described [29]. For HA, GFP and MTIP co-staining after fixation with 3% paraformaldehyde (PFA)/PBS, infected Hepa1–6 cells were permeabilised with 0.1% Triton X-100/PBS and blocked with 2% BSA/PBS. Primary antibodies were diluted in blocking solution (mouse anti-P28, 1∶1,000 [30]; rat anti-HA, 1∶200 (Roche); chicken anti-GFP, 1∶500 (Abcam); rabbit anti-MTIP, 1∶200 (gift of Dr. Matthew Jones)). Anti-rat Alexa633, anti-rabbit Alexa555 and anti-chicken Alexa488 were used as secondary antibodies together with Hoechst 33342 (all from Invitrogen), all diluted 1∶200 in blocking solution. Confocal images of ookinetes, sporozoites and liver stages were acquired with a LSM510 laser scanning confocal microscope (Zeiss). WT ookinetes, sporozoites and liver stages showed no signal for HA as control. Transmission electron microscopy (TEM) and immunogold labelling were performed as previously described [16], [31].

### Ookinete Matrigel motility assay

Ookinete cultures were added to an equal volume of Matrigel (BDbioscience) on ice, mixed thoroughly, dropped onto a slide, covered with a cover slip, and sealed with nail polish. The Matrigel was then allowed to set at 19°C for 30 minutes. After identifying a field containing ookinetes, time-lapse videos were taken (120×; 1 frame every 20 seconds, for 30 minutes) on a Leica M205A at 19°C. Movies were analyzed with Fiji and the Manual Tracking plugin (http://pacific.mpi-cbg.de/wiki/index.php/Manual_Tracking). Results are representative of three independent ookinete cultures at least.

### Sporozoite Matrigel motility assay

Isolated WT and IMC1h-KO sporozoites in RPMI (1% pen/strep) were pelleted (5 min, 21,000 g, 4°C). The supernatant was removed and the pellet was resuspended in the remaining volume, mixed with BSA (3% final) and finally mixed with Matrigel (50% final). The mixture was transferred on a microscope slide, covered with a cover slip and sealed with nail polish. Brightfield (60×; one frame every two seconds) and fluorescence (40×; two frames per second) time-lapse movies were taken at 37°C on an inverted IX81 wide field microscope with the Xcellence software (Olympus) and equipped with an ORCA-R2 camera (Hamamatsu). Movies were analyzed with Fiji and the Manual Tracking plugin (brightfield) or ToAST plugin (GFP) [17]. The range of whisker plots indicates the 2.5–97.5% percentile, the box includes 50% of all values and the horizontal line shows median values. Results are representative of sporozoites purified from three independent mosquito infections. Students t-test was performed and the threshold alpha of the p value was set to 0.01 (**).

### Hepatocyte transmigration and infection assays

Hepa 1–6 (ATCC CRL-1830) mouse hepatoma-cells were cultured in Dulbecco's Modified Eagle Medium high glucose (DMEM - Gibco/Invitrogen) with 10% FCS (Invitrogen) at 37°C with 5% CO_2_. For sporozoite infection Hepa 1–6 cells were seeded on gelatin coated (0.1% gelatin in PBS) wells of a 96 well cell culture plate (10,000 cells per well) and cultured for 24 h. For the transmigration assay cells were incubated for two hours with 2,500 and 5,000 isolated WT or IMC1h-KO sporozoites together with 1 mg/ml lysine fixable rhodamine-dextran (dextran tetramethylrhodamine; Fluoro-ruby, Molecular Probes/Invitrogen) or the dye alone (all conditions in DMEM, 10% FCS, 1% pen/strep). Cells were washed four times with complete medium and fixed with 4% PFA/PBS. For the infection assay 5,000 WT, IMC1h-KO or IMC1h-KO2 sporozoites were added to the cells in DMEM, 10% FCS, 1% pen/strep, fixed at 50hpi with 4% PFA/PBS, permeabilised/blocked with 0.2% saponin/10% FCS/1% BSA/PBS and stained with rabbit Alexa488-conjugated anti-GFP antibody (1∶200 in permeabilisation/blocking solution, Invitrogen). These protocols were adapted from [32] and [33], respectively. For both assays the nuclei were counterstained with Hoechst 33342 (Molecular Probes/Invitrogen) after fixation and images of wells were automatically acquired with a Cellomics ArrayScan VTI HCS Reader (40×; 100 fields per well) and analyzed using the SpotDetector BioApplication (all from ThermoFisher) for number of cell nuclei and dextran-positive cells for the transmigration assay. For the invasion assay, number of cell nuclei and number, area, and circularity (circularity = 4 Π[area/perimeter^2^]) of parasites were determined. Results are representative of three to six independent assays. The range of whisker plots indicates the 2.5–97.5% percentile, the box includes 50% of all values and the horizontal line shows median values. For each comparison, Students t-test was performed and the threshold alpha of the p value was set to 0.05 (*) or 0.01 (**).

## Supporting Information

Figure S1 **Generation of IMC1h-KO and IMC1h-HA parasites and their genotyping.** A. Schematic drawing illustrating the gene replacement strategy by double homologous recombination. The whole sequence of *imc1h* was replaced by a Gateway® cassette as described in [13]. B. Schematic drawing illustrating the replacement of the *imc1h* stop codon with a Gateway® cassette by double homologous recombination. C. Schematic drawing of the Gateway® cassette used in this study as described in [13]. 3′UTRs correspond to the 3′UTR of *P. berghei dhfr* and 5′ UTR corresponds to *eef1αa* 5′UTR. D. Diagnostic PCR on genomic DNA. QCR1-QCR2 primer pair amplifies unmodified *imc1h* locus only while QCR2-GW2 pair amplifies modified *imc1h* locus only. E. Southern blot analysis of XmnI-digested (XI) genomic DNA from IMC1h-KO, IMC1h-KO2 IMC1h-HA, and WT parasites. A 519 bp probe recognising the 3′UTR of *imc1h* was amplified using primer pair S1-143660 and S2-143660. F. Western blots of purified ookinetes and midgut sporozoites (40 µg of total protein) from IMC1h-HA transgenic line.
(TIF)

Figure S2 ***In vitro*** **infectivity and intracellular development of IMC1h-KO1 and IMC1h-KO2 parasites.** A. Hepa 1–6 cells invasion rate of WT and IMC1h-KO parasites at 50 hpi. Results are given as percentage of infected cells (WT1, n = 3, 136 cells; IMC1h-KO1, n = 3, 82 cells; WT2, n = 3, 205 cells; IMC1h-KO2, n = 3, 84 cells). B. Parasite area of WT and IMC1h-KO parasites in Hepa 1–6 cells 50 at hpi (WT1, n = 3, 136 cells; IMC1h-KO1, n = 3, 82 cells; WT2, n = 3, 205 cells; IMC1h-KO2, n = 3, 84 cells). C. Average circularity of WT and IMC1h-KO parasites in Hepa 1–6 cells at 50 hpi. Mutant parasites displayed a lower circularity than WT parasites indicating a more irregular and elongated shape (WT1, n = 3, 136 cells; IMC1h-KO1, n = 3, 82 cells; WT2, n = 3, 205 cells; IMC1h-KO2, n = 3, 84 cells).
(TIF)

Table S1 **Oligonucleotides used for genotyping.**
(DOCX)
