# Supplementary figures and images for: The Alveolin IMC1h Is Required for Normal Ookinete and Sporozoite Motility Behaviour and Host Colonisation in Plasmodium berghei
Source: PLoS One. 2012 Jul 23;7(7):e41409. doi: 10.1371/journal.pone.0041409 (PMC3402405; doi:10.1371/journal.pone.0041409)

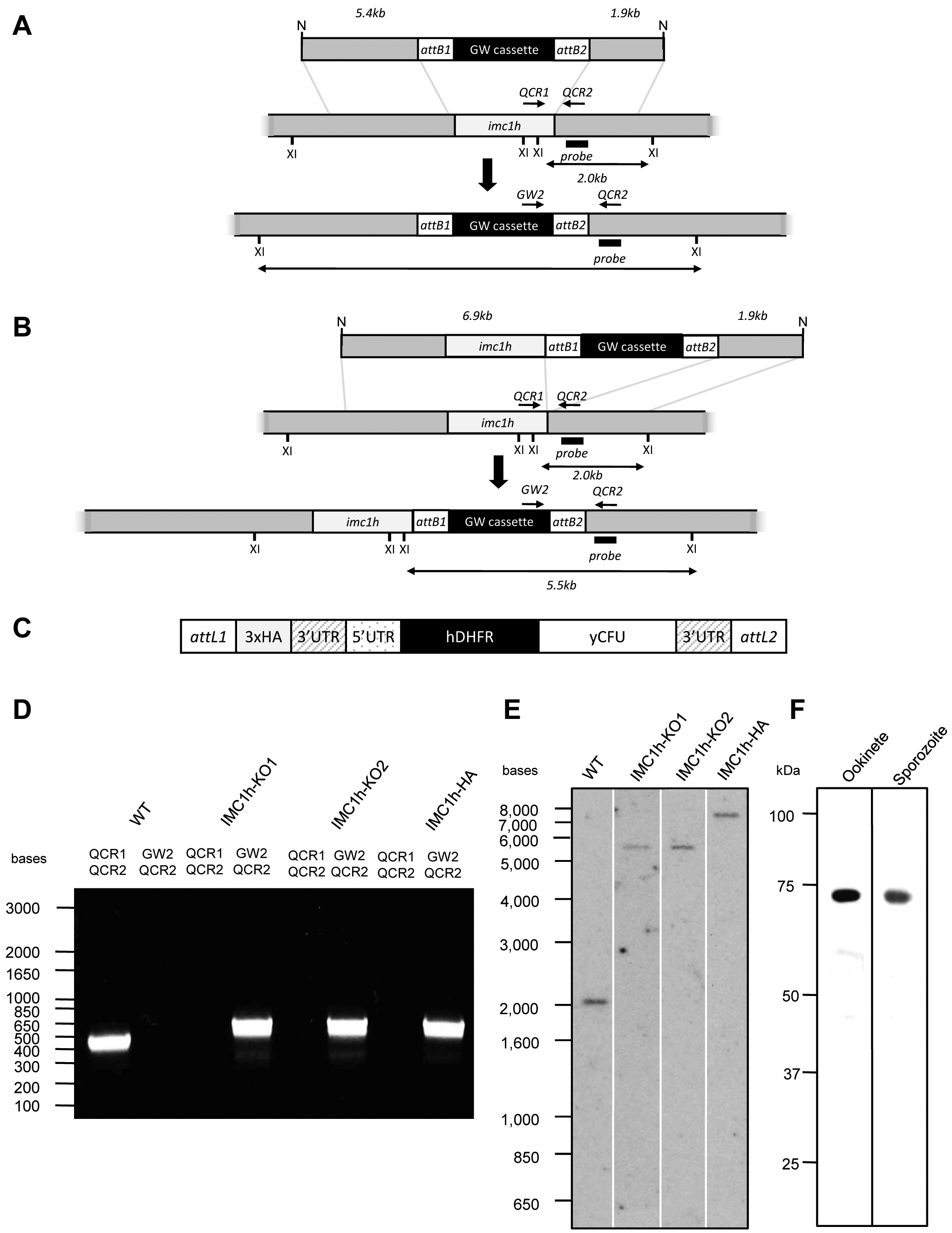

Supplement: Figure S1 — Generation of IMC1h-KO and IMC1h-HA parasites and their genotyping. A. Schematic drawing illustrating the gene replacement strategy by double homologous recombination. The whole sequence of imc1h was replaced by a Gateway® cassette as described in [13]. B. Schematic drawing illustrating the replacement of the imc1h stop codon with a Gateway® cassette by double homologous recombination. C. Schematic drawing of the Gateway® cassette used in this study as described in [13]. 3′UTRs correspond to the 3′UTR of P. berghei dhfr and 5′ UTR corresponds to eef1αa 5′UTR. D. Diagnostic PCR on genomic DNA. QCR1-QCR2 primer pair amplifies unmodified imc1h locus only while QCR2-GW2 pair amplifies modified imc1h locus only. E. Southern blot analysis of XmnI-digested (XI) genomic DNA from IMC1h-KO, IMC1h-KO2 IMC1h-HA, and WT parasites. A 519 bp probe recognising the 3′UTR of imc1h was amplified using primer pair S1-143660 and S2-143660. F. Western blots of purified ookinetes and midgut sporozoites (40 µg of total protein) from IMC1h-HA transgenic line. (TIF) [file pone.0041409.s001.tif]

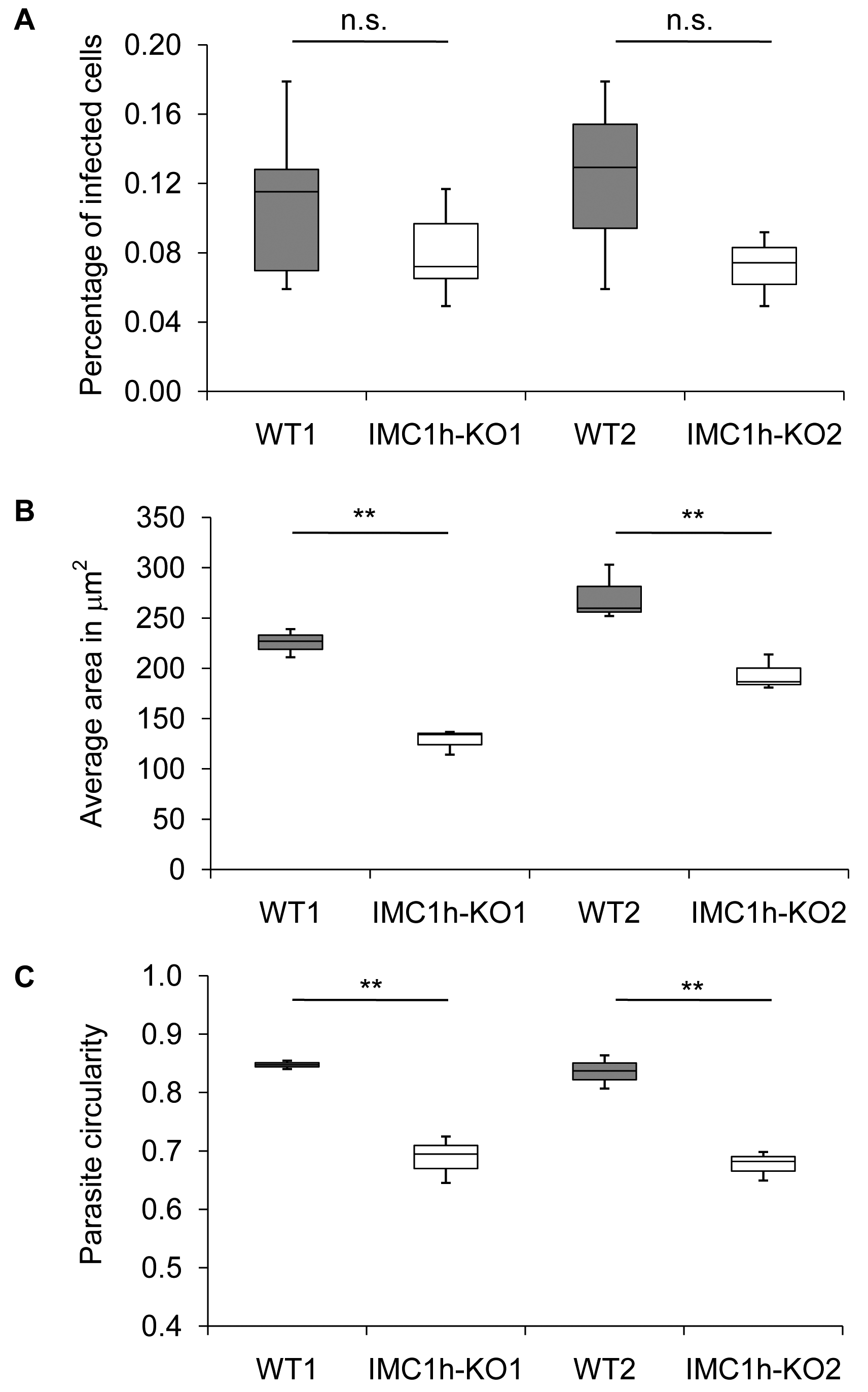

Supplement: Figure S2 — In vitro infectivity and intracellular development of IMC1h-KO1 and IMC1h-KO2 parasites. A. Hepa 1–6 cells invasion rate of WT and IMC1h-KO parasites at 50 hpi. Results are given as percentage of infected cells (WT1, n = 3, 136 cells; IMC1h-KO1, n = 3, 82 cells; WT2, n = 3, 205 cells; IMC1h-KO2, n = 3, 84 cells). B. Parasite area of WT and IMC1h-KO parasites in Hepa 1–6 cells 50 at hpi (WT1, n = 3, 136 cells; IMC1h-KO1, n = 3, 82 cells; WT2, n = 3, 205 cells; IMC1h-KO2, n = 3, 84 cells). C. Average circularity of WT and IMC1h-KO parasites in Hepa 1–6 cells at 50 hpi. Mutant parasites displayed a lower circularity than WT parasites indicating a more irregular and elongated shape (WT1, n = 3, 136 cells; IMC1h-KO1, n = 3, 82 cells; WT2, n = 3, 205 cells; IMC1h-KO2, n = 3, 84 cells). (TIF) [file pone.0041409.s002.tif]
