# Supplementary material for: The Alveolin IMC1h Is Required for Normal Ookinete and Sporozoite Motility Behaviour and Host Colonisation in Plasmodium berghei
Source: PLoS One. 2012 Jul 23;7(7):e41409. doi: 10.1371/journal.pone.0041409 (PMC3402405; doi:10.1371/journal.pone.0041409)
Supplement: Table S1 — Oligonucleotides used for genotyping. (DOCX) [file pone.0041409.s003.docx]

**Table S1. Oligonucleotides used for genotyping.**

| Primer Name | Sequence |
| --- | --- |
| S1-143660 | ACAGAACATATGCTCAAGC |
| S2-143660 | ATGACCCAAACAAAATAGC |
| QCR1-143660 | GACAAAATAATTCGAATTTG |
| QCR2-143660 | GCTTGAGCATATGTTCTGT |
| GW2 | GTATCTGTTCCAGGGTTG |
